# Longer DNA exhibits greater potential for cell-free gene expression

**DOI:** 10.1038/s41598-021-91243-x

**Published:** 2021-06-03

**Authors:** Takashi Nishio, Yuko Yoshikawa, Kenichi Yoshikawa, Shin-ichi Sato

**Affiliations:** 1 Faculty of Life and Medical Sciences, Doshisha University, Kyoto, 610-0394 Japan; 2 Institute for Chemical Research, Kyoto University, Kyoto, 611-0011 Japan

**Keywords:** Biophysics, Molecular biology

## Abstract

Cell-free gene expression systems have been valuable tools for understanding how transcription/translation can be regulated in living cells. Many studies have investigated the determining factors that affect gene expression. Here we report the effect of the length of linearized reporter DNAs encoding the firefly luciferase gene so as to exclude the influence of supercoiling. It is found that longer DNA molecules exhibit significantly greater potency in gene expression; for example, the expression level for DNA with 25.7 kbp is 1000-times higher than that for DNA of 1.7 kbp. AFM observation of the DNA conformation indicates that longer DNA takes shrunken conformation with a higher segment density in the reaction mixture for gene expression, in contrast to the stiff conformation of shorter DNA. We propose an underlying mechanism for the favorable effect of longer DNA on gene expression in terms of the enhancement of access of RNA polymerase to the shrunken conformation. It is expected that the enhancement of gene expression efficiency with a shrunken DNA conformation would also be a rather general mechanism in living cellular environments.

## Introduction

Gene expression in living cells is strictly self-regulated to ensure that the correct amounts of proteins are made at the most appropriate timing and location for maintaining cellular homeostasis. Gene regulation can occur at any point in gene expression, from the start of the transcription phase to the translation phase. Due to the complexity of gene regulation, unveiling the complete mechanism of gene regulation has been a long-standing quest. To elucidate the complex mechanism of gene regulation in living cellular systems, it is necessary to closely investigate each phase of gene expression and also to shed light on the cooperative effects between transcription and translation. One of the best methods for such an investigation may be the use of a coupled transcription/translation system which allows artificial control of the experimental conditions in vitro^1–3^. The methodology of cell-free gene expression developed during the last couple of decades in molecular biology is expected to provide a useful experimental system for the purpose to gain deeper insights into the coupled transcription/translation dynamics^1–3^. In this regard, cell-free gene expression systems are one of the most widely used techniques in molecular biology^1–3^. The efficiency of cell-free gene expression is known to be affected by various factors such as vector type^4–7^, DNA topology^4–7^, and reaction components^8–11^, as well as the template DNA concentration^12–14^. It is noted that there exists a longstanding controversy regarding the topological effect of supercoiling, or linking number, in circular DNAs on their genomic activity^4–7^. Although these factors have been studied extensively, little is known about how the length of the DNA template influences gene expression. The aforementioned complex factors have made it difficult to investigate the impact of the DNA length on gene regulation, which has been an unexplored topic. In the present study, we evaluated the efficiency of protein translation, focusing on the effect of DNA templates of different lengths on cell-free protein synthesis using a luciferase assay. To exclude the influence of supercoiling, we used linear DNA molecules (PCR amplicons or linearized by restriction enzyme) as a template.

## Results

**Figure 1:**
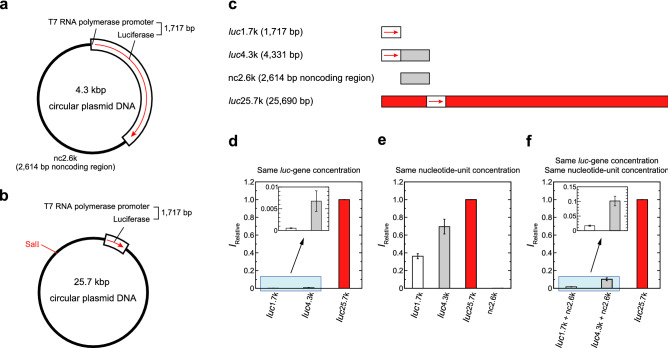
Effects of various factors on the efficiency of gene expression. (**a**,**b**) Schematic diagram of the parental 4.3-kbp and 25.7-kbp plasmid DNAs. The white box on the plasmid DNA represents a firefly luciferase reporter gene with a T7 promoter. The red arrow in the white box shows the direction of reporter gene transcription. (**c**) DNA fragments resulting from Sal I digestion of the parental 25.7-kbp plasmid or PCR amplification. Sal I yields a single 25.7-kbp linear reporter gene. A 1.7-kbp reporter gene, a 4.3-kbp reporter gene, and a 2.6-kbp non-coding DNA fragment were prepared by standard PCR. The 25.7-kbp linear reporter gene and the 2.6-kbp non-coding DNA region are colored in red and gray, respectively. (**d**–**f**) Efficiency, *I*, of the coupled transcription/translation reaction of *luc*-gene, as evaluated from the change of luminescence intensity. The same *luc*-gene concentration (0.12 μM, **d**), the same nucleotide-unit concentration (1.8 μM, **e**), or a combination of both (the same *luc*-gene and nucleotide-unit concentration) (0.12 μM and 1.8 μM, **f**) of reporter genes. In (**d–f**), the longitudinal axis shows the relative luminescence intensity normalized to that of *luc*25.7 k. The relative protein expression levels with *luc*25.7 k are presented as means ± SD of at least five independent experiments.

**Figure 2:**
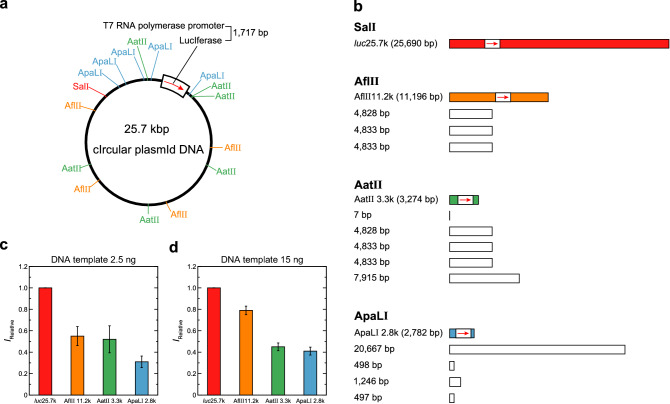
Nucleotide-length dependency of the coupled transcription/translation of the *luc*-gene. (**a**) Schematic diagram of the parental 25.7-kbp plasmid DNA with restriction enzyme digestion sites. The white box on the plasmid DNA represents a firefly luciferase reporter gene with a T7 promoter. The red arrow in the white box shows the direction of reporter gene transcription. (**b**) DNA fragments resulting from enzyme digestion reactions of the parental plasmid. Sal I yields a single 25.7-kbp linear reporter gene. Afl II, Aat II and ApaL I generate a 11.2-kbp reporter gene and three kinds of 4.8-kbp non-coding DNA fragments, a 3.3-kbp reporter gene and five kinds of non-coding DNA fragments (7-bp, three 4.8-kbp and 7.9-kbp lengths), and a 2.8-kbp reporter gene and four kinds of non-coding DNA fragments (20.7-kbp, 1.2-kbp and two 0.5-kbp lengths), respectively. The reporter genes in the DNA fragments are colored red for Sal I, orange for Afl II, green for Aat II and light blue for ApaL I treatments, respectively. (**c**,**d**) Efficiency, *I*, of the coupled transcription/translation reaction of *luc*-gene, as evaluated from the luminescence intensity. 2.5 ng (**c**) and 15 ng (**d**) of digested DNA mixtures were used as DNA templates for the reaction. In (**c**,**d**), the longitudinal axis shows the relative luminescence intensity normalized to that of *luc*25.7k. The relative protein expression levels with *luc*25.7k are presented as means ± SD of at least five independent experiments.

We compared the levels of gene expression of reporter DNAs encoding the firefly luciferase gene (*luc*-gene) with different lengths of 1717 bp, 4331 bp, and 25,690 bp; hereafter we may call as *luc*1.7k, *luc*4.3k and *luc*25.7k, respectively (Fig. 1a–c and Fig. S1 in the Supplementary Information). We used three different conditions: the same *luc*-gene concentration (Fig. 1d), the same nucleotide-unit concentration (Fig. 1e), and a combination of both (the same *luc*-gene and nucleotide-unit concentration adjusted by the addition of noncoding DNA, *nc*2.6k) (Fig. 1f). The expression level from *luc*25.7k is 1000-times higher than that with *luc*1.7k, indicating that the protein yield per target gene is increased by 1000-times of magnitude for a longer template, *luc*25.7k (Fig. 1d). Under the condition of the same nucleotide-unit concentration, where the concentrations of the targets are inversely proportional to the length of DNA molecules (Fig. 1e), the expression level from *luc*25.7k is highest despite the fact that *luc*25.7k has the fewest targets. Interestingly, in a comparison of *luc*1.7k and *luc*4.3k, *luc*4.3k induced a 7-times (Fig. 1d) and 2-times (Fig. 1e) increase in the gene expression level, respectively. These findings indicate that deletion of a 2.6-kb noncoding region results in a lower protein yield, i.e., the magnitude of gene expression increases when the luciferase-coding region is connected to the noncoding region. To validate this result, the level of gene expression was compared under the condition that both the amount of *luc*-gene and the nucleotide concentration were unified by the addition of extra *nc*2.6k to the reaction buffer (Fig. 1f). It is shown that the addition of *nc*2.6k to *luc*1.7k and *luc*4.3k caused an order of magnitude greater protein yields (Fig. 1d,f), suggesting the intrinsic effect of nucleotide concentration on gene expression efficiency. Nevertheless, it is clear that the expression efficiency per target is enhanced significantly when the length of the template DNA molecule is longer (Fig. 1f).

The above results clearly indicate that the level of gene expression markedly increased in response to an increase in the length of templates, in addition to the effect of nucleotide concentration in the reaction buffer solution. It is revealed that the potency of expression is in the order of *luc*25.7k > *luc*4.3k > *luc*1.7k under all conditions tested here (Fig. 1). It depends not on the number of targets, but rather on the length of the template DNA. These results also suggest that the noncoding region in reporter DNAs is involved in the regulation of gene expression.

The design of the linear DNAs shown in Fig. 1 may not be convincing enough to investigate the influence of DNA length on gene expression as the non-specific sequences of both up and down streams of the T7 RNA promoter are different in each gene. To address the concern in experimental design, we next designed an in vitro translation assay with four kinds of reporter genes of different nucleotide lengths. The four reporter DNAs encoding firefly luciferase with a T7 promoter were obtained from an identical 25.7-kbp plasmid DNA cleaved with restriction enzyme Sal I, Afl II, Aat II, or ApaL I, respectively (Fig. 2a). Sal I yields a single 25.7-kbp linear reporter gene, and the other enzymes generate reporter genes of different lengths with non-coding DNA fragments (Fig. 2b). The resulting DNA mixtures with reporter genes of different lengths (ApaLI 2.8k, AatII 3.3k, AflII 11.2k and *luc*25.7k) were used to evaluate their translational efficiency as DNA templates for continuous coupled transcription/translation. As expected, reporter gene expression increased in a nucleotide-length-dependent manner on the DNA templates while the amount and composition of nucleotides remained identical in each reaction (Fig. 2c,d). These results strongly suggest that DNA length positively affects gene activities in a cell-free gene expression system.

**Figure 3:**
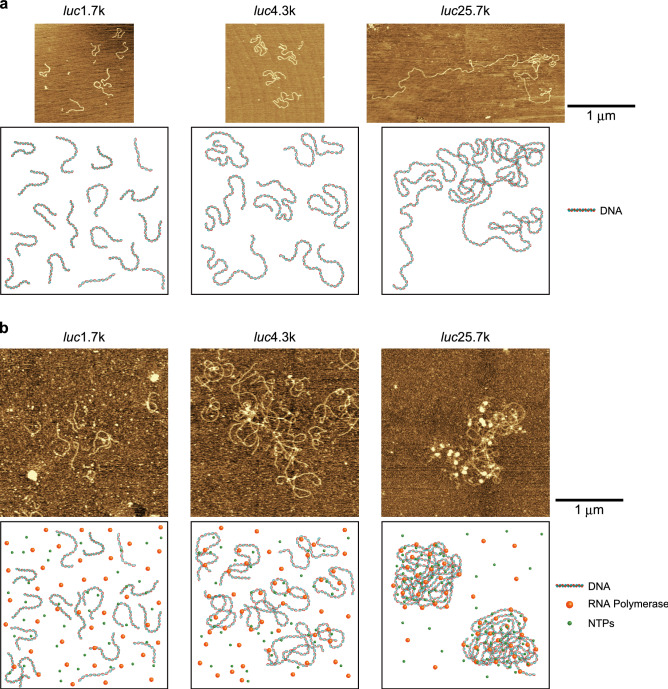
AFM images of reporter DNAs with different lengths together with the schematic representations. (**a**) AFM images of each linear reporter DNAs in 10 mM Tris–HCl buffer (pH7.5). (**b**) AFM images of each linear reporter DNA in a 0.05% reaction mixture for in vitro gene expression.

To determine the effect of reporter gene size on gene expression activity, single-molecule observations of each linear reporter gene were performed by AFM. The AFM images of each DNA in 10 mM Tris–HCl buffer (pH7.5) revealed a linearized structure, indicating that DNA molecules tend to exhibit a more winding structure as their length increases (Fig. 3a). Notably, in a 0.05% reaction mixture for in vitro gene expression, *luc*25.7k formed a shrunken structure with a higher density of DNA segments than that of *luc*4.3k and *luc*1.7k (Fig. 3b and Fig. S2 in the Supplementary Information). Based on these observations, we consider that the sliding and hopping behaviors of RNA polymerase, which have been studied extensively, is enhanced with longer DNA. In addition, proteins coexisting in the gene expression reaction buffer tend to localize onto the shrunken conformation for the larger DNA molecules, suggesting that the effective affinity of RNA polymerase for DNA is enhanced in longer DNA (see the schematic representations on the lower pictures in Fig. 3b). In the following discussion, we may argue the mechanism on the promotion of gene expression in relation to the physico-chemical characteristics of longer DNA molecules.

Cells regulate gene expression to maintain cellular functions. Gene expression starts with transcription and is continuously followed by translation events. Hence, the initiation of transcription is an efficient point for controlling protein production. In the present study, we demonstrated that DNA length is a crucial factor for gene expression by using a coupled transcription/translation system. The positive effects of DNA length on gene expression efficiency can mainly be explained by increased transcription activity, while mRNA stability, nucleases’ activity, and template degradation should also be taken into account. In fact, it appeared that mRNA was slightly less stable than ribosomal RNA when the stability of mRNA in reticulocyte cell lysates was examined by RT-qPCR (Table S1 in the Supplementary Information). Meanwhile, it is noted that the lengths and stabilities of transcripts from each reporter gene in Fig. 2 are the same as those of nucleotide from the T7 promoter to its terminator. It is thus regarded that the translation phase is not involved in controlling gene expression in the present experiments in a cell-free system.

## Discussion

A plausible mechanism for the promoting effect of DNA length on transcriptional efficiency may be represented by the difference in the conformation of DNA molecules. It is getting clearer that the higher-order structure of the template DNA molecule plays an important role as a controlling factor on the activity of gene expression. To date, not a number of studies have reported that transcriptional condensates with genomic DNA serve as regulatory hubs in gene expression^15–24^. As for the characteristic property of the high-order structure on DNA, it has been established that giant DNA above the size of several tens kbp undergoes a large discrete transition from an elongate coil state into a tightly compact state, accompanied by the increase of concentration of various polycationic species such as polyamines^25,26^. It is known that transcriptional activity is completely inhibited in the tightly compact state^27,28^. Recently, it has been found that gene expression is promoted at the lower concentrations of polyamines than the concentration that causes compaction^9^. That is, maximum expression is generated in the shrunken state where the density of DNA segments is higher than in the elongated coil state by avoiding tight packing. Inspection for the actual structure of the shrunken conformation has indicated enhancement of parallel orientation between DNA segments in a shrunken state of long DNA^9,10,29^. In other words, the basic property of the alignment of DNA segments resembles that of liquid crystals, where rod-like elements prefer to be arranged in parallel under weak repulsive interactions^9^.

If we consider the above-mentioned effect of the shrunken conformation in the reaction mixture on gene expression, we can expect that formation of the shrunken conformation with a preference for parallel alignment accelerates gene expression and that such a promoting effect would be caused by the greater ability of RNA polymerase to access shrunken DNA. We denote the length of template DNA as *L*, corresponding to the ‘contour length’ in the definition of polymer physics^30^. For simplicity, if we assume that the average distance between DNA segments is nearly constant, the volume, *v*, of a DNA molecule in the shrunken state would exhibit a scaling relationship with *L* as $v \sim L$, which is different from a polymer chain in a good solvent, $v \sim {L}^\frac{9}{5}$, and an ideal chain, $v \sim {L}^\frac{3}{2}$^30^. The preferential binding of RNA polymerase together with the enhanced location of NTPs would be effective in the inner volume of the shrunken state. Thus, we may consider that the effective volume of the active region of translation is given by subtracting the infectivity on the surface region: ${v}_{E} \sim (L-{\alpha L}^\frac{2}{3})$, where we used an approximation of scaling relationships; $v\sim L,\left[\mathrm{Surface Area}\right]\sim {L}^\frac{2}{3}$, and $\alpha$ is a positive constant. Now, we may evaluate the relative activity of gene expression as in the case of Fig. 1e. By taking the variables *n* (number density of DNA) and *C* (density of DNA in base pair units), the relationship is obtained as in $nL=C.$ From these relationships, the total active volume of DNA molecules is deduced to be ${V}_{E }\sim n{v}_{E} \sim \frac{C}{L}\left(L- \alpha {L}^\frac{2}{3}\right)=C(1-\alpha {L}^{-\frac{1}{3}})$. Thus, the value ${L}^{-\frac{1}{3}}$ corresponds to the degree of the decrease in gene expression, which is 2.5, 2.0 and 1.0 for template DNAs of *luc*1.7k, *luc*4.3k and *luc*25.7k, respectively. By taking the gene expression of *luc*1.7k as a control, the increase in activity is estimated from the calculations of “2.5–2.0 = 0.5” and “2.5–1.0 = 1.5”, i.e., 0.5 and 1.5, for *luc*4.3k and *luc*25.7k, respectively. These expected values of the increase in genetic activities correspond to the observed experimental trend, despite the simplicity of the theoretical argument.

In the present article, we found that longer DNA enhances the efficiency of gene expression. It is known that DNA molecules much larger than the persistence length (150–200 bp) behave as semiflexible polymer chains^31–33^. Here, it has become clear that such relatively long DNA molecules exhibit a shrunken conformation in reaction buffer, which increases the opportunity for encountering RNA polymerase and NTPs due to an increase in their effective concentration around DNA. Although the discovery of the size effect of DNA on gene expression is still in a preliminary stage, it would be promising to extend this insight by shedding light on the actual mechanisms of gene regulation in living cells through extensive studies for longer DNA molecules with the sizes of Mbp–Gbp. It would also be interesting to examine the effect of DNA length on the activity of other enzymes, such as restriction enzymes, DNA repair enzymes, DNA polymerase, etc.

## Methods

### Preparation of linear reporter genes by PCR

A 4.3-kbp plasmid DNA (Luciferase T7 Control DNA: 4331 bp) was purchased from Promega (Madison, WI, USA). A 1.7-kbp linear reporter DNA, a 4.3-kbp linear reporter DNA and a 2.6-kbp linear non-coding DNA were amplified by PCR using the following primers: for the 1.7-kbp linear DNA (forward 5′-TAATACGACTCACTATAGGGAGACC-3′; reverse 5′-CAATTTGGACTTTCCGCCCTTC-3′); for the 4.3-kbp linear DNA (forward 5′-TAATACGACTCACTATAGGGAGACC-3′; reverse 5′-ATTTCGATAAGCCAGCTGC-3′); and for the 2.6-kbp non-coding DNA (forward 5′-CTGTATTCAGCGATGACGAAATTC-3′; reverse 5′-ATTTCGATAAGCCAGCTGC-3′). 1 ng of the 4.3-kbp parental plasmid was used as a template for PCR. PCR was performed with KOD FX neo (Toyobo, Tokyo, Japan) using a Thermal Cycler Dice (TP600) (Takara Bio Inc, Shiga, Japan), according to the manufacturer’s protocol. The PCR products were precipitated with 0.3 M sodium acetate/ethanol, washed with 70% ethanol, and dried. The DNA pellets were dissolved in 50 μL of TE buffer (pH 8.0) and stored at − 20 °C until use. Each template DNA was analyzed using a DNA ladder marker (1 kbp DNA Ladder One, Nacalai Tesque, Kyoto, Japan) by standard 1% agarose gel electrophoresis (Fig. S1b,d in the Supplementary Information).

### Preparation of linear reporter genes by restriction enzyme treatment

A parental 25.7-kbp plasmid DNA was purchased from Kazusa Genome Technologies (Chiba, Japan). The whole sequence of the plasmid is shown in Supplementary Sequence 1. Restriction enzymes Sal I, Afl II, Aat II and ApaL I were purchased from Takara Bio Inc. 2 μg of the 25.7-kbp plasmid was cleaved with 30 U of each restriction enzyme in 100 μL of T buffer (Takara Bio Inc) containing 0.1% BSA. The reaction mixtures were incubated for 120 min at 37 °C. After enzymatic digestion, the DNA mixtures were precipitated with 0.3 M sodium acetate/ethanol, washed with 70% ethanol, and dried. The DNA pellets were dissolved in 50 μL of TE buffer (pH 8.0) and stored at − 20 °C until use. Each digested DNA was analyzed using a DNA ladder marker (1 kbp DNA Ladder One, Nacalai Tesque, Kyoto, Japan) by standard 1% agarose gel electrophoresis (Fig. S1c,d in the Supplementary Information).

### AFM observation

For AFM imaging using an SPM-9700 (Shimadzu, Kyoto, Japan) with a High-Throughput Scanner (Shimadzu, Kyoto, Japan), 0.3 μM DNA was dissolved in 10 mM Tris–HCl buffer (pH 7.5) including 10 μM spermidine or in 0.05% TnT Quick Master Mix (in vitro gene expressions reaction mixture). The DNA solutions were transferred onto a freshly cleaved mica surface and then incubated for 10–30 min at room temperature (24 °C). Subsequently, the samples were rinsed with ultra-pure water, dried with nitrogen gas and imaged by AFM. All measurements were performed in air using the tapping mode. The cantilever, OMCL-AC200TS-C3 (Olympus, Tokyo, Japan), was 200 μm long with a spring constant of 9–20 N/m. The scanning rate was 2 Hz and images were captured using the height mode in a 512 × 512 pixel format. The obtained images were plane-fitted and flattened by the computer program supplied with the imaging module.

### Luciferase reporter assay

Cell-free in vitro transcription/translation of *Luc*-gene was performed with a TnT T7 Quick Coupled Transcription/Translation System (Promega) according to the manufacturer's instructions and previous reports^9–11^. Reaction mixtures containing the reporter DNAs were incubated for 90 min at 30 °C on a Dry Thermo Unit (TAITEC, Saitama, Japan). The expression levels of luciferase were evaluated following the addition of luciferin as a luciferase substrate (Luciferase Assay Reagent, Promega) by detecting the emission intensity at around 565 nm using a luminometer (MICROTEC Co., Chiba, Japan).

## Supplementary Information

Supplementary Information 1.

## Data Availability

The data analyzed in this paper are available from the corresponding author on reasonable request.
