# Supplementary material for: Longer DNA exhibits greater potential for cell-free gene expression
Source: Sci Rep. 2021 Jun 3;11:11739. doi: 10.1038/s41598-021-91243-x (PMC8175755; doi:10.1038/s41598-021-91243-x)
Supplement: Supplementary file 1 — Supplementary Information 1. [file 41598_2021_91243_MOESM1_ESM.pdf]

Supplementary Information for

Longer DNA exhibits greater potential for cell-free gene expression

Takashi Nishio[1], Yuko Yoshikawa[1], Kenichi Yoshikawa[1] & Shin-ichi Sato[2,*]

[1]Faculty of Life and Medical Sciences, Doshisha University, Kyoto, 610-0394, Japan.

[2]Institute for Chemical Research, Kyoto University, Kyoto, 611-0011, Japan.

*e-mail: ssato@scl.kyoto-u.ac.jp

1. AFM images and gel electrophoresis analysis

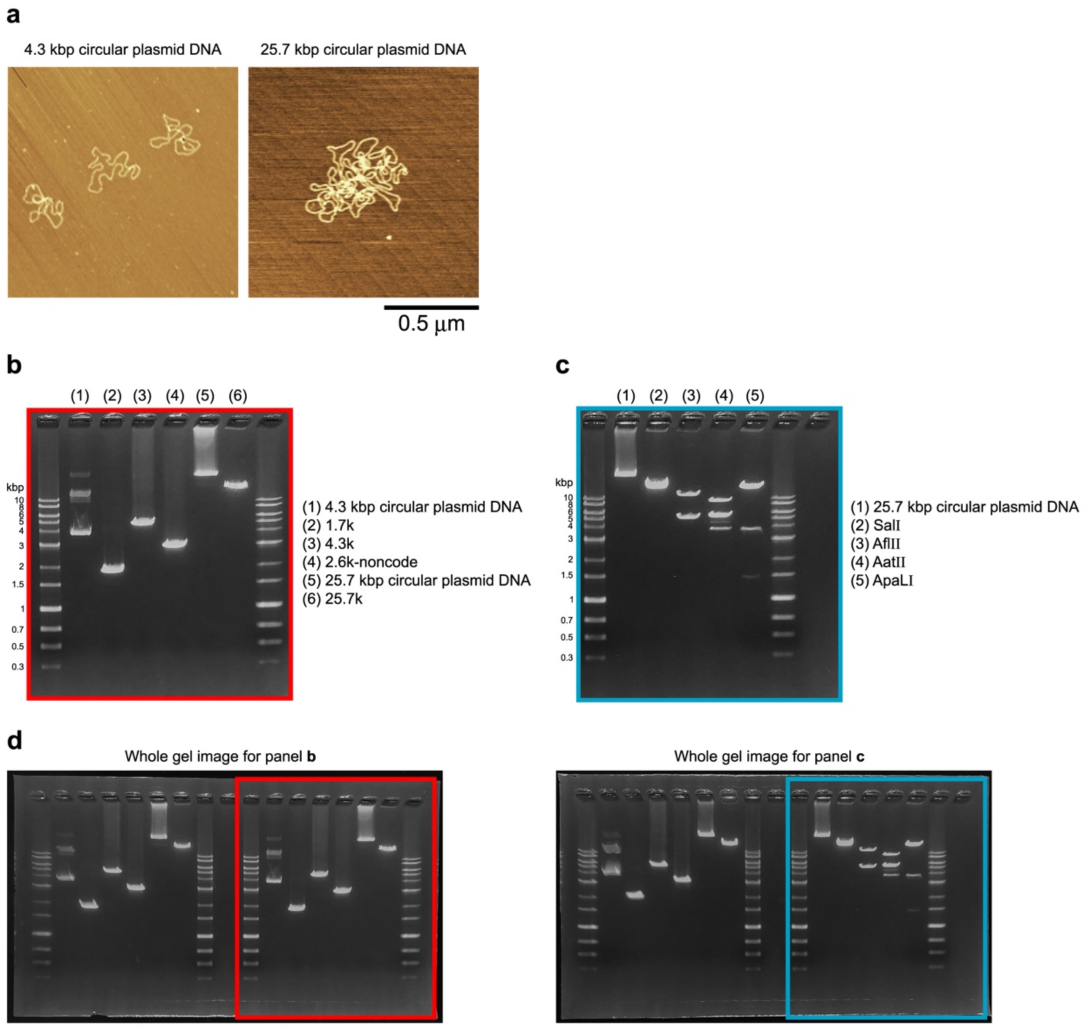

Figure S1. **a**, AFM images of the parental 4.3k and 25.7k plasmid DNAs. **b**, Gel electrophoresis analysis of parental 4.3k and 25.7k plasmid DNAs and linearized DNAs obtained from each of these circular plasmid DNAs. **c**, Gel electrophoresis analysis of reporter genes of different lengths with non-coding DNA fragments generated by each restriction enzyme. **d**, Whole gel images for panel **b** and **c**. The cropped areas are indicated red and blue square for each.

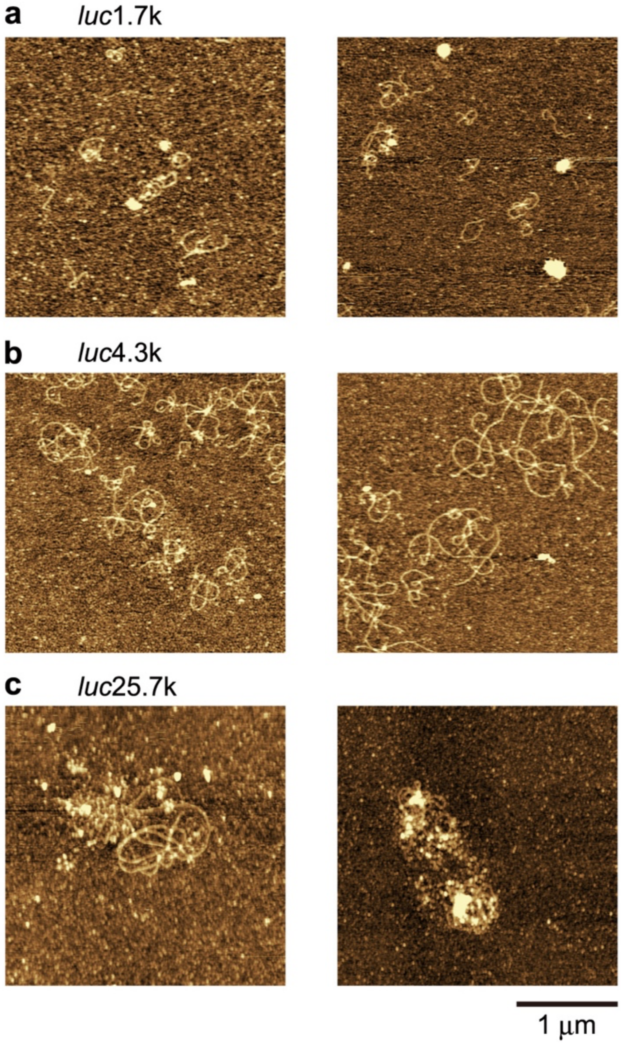

Figure S2. Additional AFM images of the linear reporter genes (*luc*1.7k (**a**), *luc*4.3k (**b**), and *luc*25.7k (**c**)) in a 0.05% reaction mixture for *in vitro* gene expression.

2. Evaluation of mRNA stability

Method A 1.7 kb luciferase mRNA was mixed with reticulocyte cell lysates in a TnT T7 Quick Coupled Transcription/Translation System (Promega). After 0, 30, 60 and 90 mins incubation at 30 °C, the total RNAs were isolated using ISOGEN (Nippon Gene Co., Ltd.), according to the manufacturer's protocol. First strand cDNAs were reverse-transcribed from 200 ng of the RNA samples, using a random primer and PrimeScript™ II 1st strand cDNA Synthesis Kit (Takara Bio Inc.), according to the manufacturer's protocol. The cDNAs were subjected to qPCR using a pair of luciferase primers for quantification of luciferase mRNA [5'-GCG CGG AGG AGT TGT GTT-3' and 5'-TCT GAT TTT TCT TGC GTC GAG TT-3'], and a primer pair for quantification of S18 rRNA [5'-AAA CGG CCA CCA CAT CCA AG-3' and 5'-CAA TTG CAG GGC TCT CGA AG-3'].

Table S1. RT-qPCR quantification of mRNA stability. Time course mRNA measurement for quantification of mRNA stability in cell lysates was performed by RT-qPCR. The amount of luciferase mRNA and 18S rRNA in each sample was normalized to no incubated samples, respectively: $\Delta C_T = C_T$ (30, 60, and 90 min) – C_T (0 min). $\Delta\Delta C_T$ values were determined by subtracting the ΔC_T values for S18S rRNA from the ΔC_T values for luciferase mRNA. Results are for two independent experiments.

Templates	Time (min)	C_T	ΔC_T*	$\Delta\Delta C_T$**
Luc mRNA	0	12.1 ± 0.7	-	-
	30	11.7 ± 0.0	-0.4	-0.1
	60	12.3 ± 0.1	0.2	0.5
	90	12.8 ± 0.1	0.7	1.0
18S rRNA	0	17.2 ± 0.5	-	-
	30	16.7 ± 0.1	-0.3	-
	60	16.9 ± 0.1	-0.3	-
	90	16.9 ± 0.2	-0.3	-

*$\Delta C_T = C_T$ (incubate time (min))-C_T (0 min)

**$\Delta\Delta C_T = \Delta C_T$ (Luc mRNA)-ΔC_T (18S rRNA)

3. Whole base sequence of 25.7 kbp (25,690) plasmid DNA.

Supplementary Sequence 1. Whole sequence of 25.7 kbp (25,690 bp) circular plasmid DNA. Color coding is as follows:

Underlined italic: T7 RNA polymerase promoter 881-900 (20 bp)

Bold: Luciferase gene 948-2,597 (1,650 bp)

TCGCGCGTTTCGGTGATGACGGTGAAAACCTCTGACACATGCAGCTCCCGGAGACGGTCACAGCTTGTCTGTAAGC

GGATGCCGGGAGCAGACAAGCCCGTCAGGGCGCGTCAGCGGGTGTTGGCGGGTGTCGGGGCTGGCTTAACTATG

CGGCATCAGAGCAGATTGTACTGAGAGTGCACCATAAAATTGTAAACGTTAATATTTTGTTAAAATTCGCGTTAAATTTT

TGTTAAATCAGCTCATTTTTTAACCAATAGGCCGAAATCGGCAAAATCCCTTATAAATCAAAAGAATAGCCCGAGATAG

GGTTGAGTGTTGTTCCAGTTTGGAACAAGAGTCCACTATTAAAGAACGTGGACTCCAACGTCAAAGGGCGAAAAAC

CGTCTATCAGGGCGATGGCCCACTACGTGAACCATCACCCAAATCAAGTTTTTTGGGGTCGAGGTGCCGTAAAGCA

CTAAATCGGAACCCTAAAGGGAGCCCCCGATTTAGAGCTTGACGGGGAAAGCCGGCGAACGTGGCGAGAAAGGAA

GGGAAGAAAGCGAAAGGAGCGGGCGCTAGGGCGCTGGCAAGTGTAGCGGTCACGCTGCGCGTAACCACCACACC

CGCCGCGCTTAATGCGCCGCTACAGGGCGCGTACTATGGTTGCTTTGACGTATGCGGTGTGAAATACCGCACAGAT

GCGTAAGGAGAAAATACCGCATCAGGCGCCATTCGCCATTCAGGCTGCGCAACTGTTGGGAAGGGCGATCGGTGC

GGGCCTCTTCGCTATTACGCCAGCTGGCGAAAGGGGGATGTGCTGCAAGGCGATTAAGTTGGGTAACGCCAGGGT

TTTCCCAGTCACGACGTTGTAAAACGACGGCCAGTGCCAAGCTG*TAATACGACTCACTATAGGG*AGACCCAAGCTTA

TGCATGCGGCCGCATCTAGAGGGCCCGGATCCAA**ATGGAAGACGCCAAAAACATAAAGAAAGGCCCGGCGCCAT**

TCTATCCTCTAGAGGATGGAACCGCTGGAGAGCAACTGCATAAGGCTATGAAGAGATACGCCCTGGTTCCTGGAA

CAATTGCTTTTACAGATGCACATATCGAGGTGAACATCACGTACGCGGAATACTTCGAAATGTCCGTTCGGTTGGC

AGAAGCTATGAAACGATATGGGCTGAATACAAATCACAGAATCGTCGTATGCAGTGAAAACTCTCTTCAATTCTTTA

TGCCGGTGTTGGGCGCGTTATTTATCGGAGTTGCAGTTGCGCCCGCGAACGACATTTATAATGAACGTGAATTGCT

CAACAGTATGAACATTTCGCAGCCTACCGTAGTGTTTGTTTCCAAAAAGGGGTTGCAAAAAATTTTGAACGTGCAA

AAAAAATTACCAATAATCCAGAAAATTATTATCATGGATTCTAAAACGGATTACCAGGGATTTCAGTCGATGTACAC

GTTCGTCACATCTCATCTACCTCCCGGTTTTAATGAATACGATTTTGTACCAGAGTCCTTTGATCGTGACAAAACAA

TTGCACTGATAATGAATTCCTCTGGATCTACTGGGTTACCTAAGGGTGTGGCCCTTCCGCATAGAACTGCCTGCGT

CAGATTCTCGCATGCCAGAGATCCTATTTTTGGCAATCAAATCATTCCGGATACTGCGATTTTAAGTGTTGTTCCATT

CCATCACGGTTTTGGAATGTTTACTACACTCGGATATTTGATATGTGGATTTCGAGTCGTCTTAATGTATAGATTTGA

AGAAGAGCTGTTTTTACGATCCCTTCAGGATTACAAAATTCAAAGTGCGTTGCTAGTACCAACCCTATTTTCATTCT

TCGCCAAAAGCACTCTGATTGACAAATACGATTTATCTAATTTACACGAAATTGCTTCTGGGGGCGCACCTCTTTCG

AAAGAAGTCGGGGAAGCGGTTGCAAAACGCTTCCATCTTCCAGGGATACGACAAGGATATGGGCTCACTGAGAC

TACATCAGCTATTCTGATTACACCCGAGGGGGATGATAAACCGGGCGCGGTCGGTAAAGTTGTTCCATTTTTTGAA

GCGAAGGTTGTGGATCTGGATACCGGGAAAACGCTGGGCGTTAATCAGAGAGGCGAATTATGTGTCAGAGGACC

TATGATTATGTCCGGTTATGTAAACAATCCGGAAGCGACCAACGCCTTGATTGACAAGGATGGATGGCTACATTCT

GGAGACATAGCTTACTGGGACGAAGACGAACACTTCTTCATAGTTGACCGCTTGAAGTCTTTAATTAAATACAAAG

GATATCAGGTGGCCCCCGCTGAATTGGAATCGATATTGTTACAACACCCCAACATCTTCGACGCGGGCGTGGCAG
GTCTTCCCGACGATGACGCCGGTGAACTTCCCGCCGCCGTTGTTGTTTTGGAGCACGGAAAGACGATGACGGAA
AAAGAGATCGTGGATTACGTCGCCAGTCAAGTAACAACCGCGAAAAAGTTGCGCGGAGGAGTTGTGTTTGTGGA
CGAAGTACCGAAAGGTCTTACCGGAAAACTCGACGCAAGAAAAATCAGAGAGATCCTCATAAAGGCCAAGAAGG
GCGGAAAGTCCAAATTGTAAAATGTAACTGTATTCAGCGATGACGAAATTCTTAGCTATTGTAATCCTCCGAGGGGGC
GAGCTCCCAAAAAAAAAAAAAAAAAAAAAAAAAAAAAACCGAATTGGCATCACCGGCGCCACAGGTGCGGTTGCTG
GCGCCTATATCGCCGACATCACCGATGGGGAAGATCGGGCTCGCCACTTCGGGCTCATGAGCGCTTGTTTCGGCGT
GGGTATGGTGGCAGGCCCCGTGGCCGGGGGACTGTTGGGCGCCATCTCCTTGCATGCACCATTCCTTGCGGCGG
CGGTGCTCAACGGCCTCAACCTACTACTGGGCTGCTTCCTAATGCAGGAGTCGCATAAGGGAGAGCGTCGAATGGT
GCACTCTCAGTACAATCTGCTCTGATGCCGCATAGTTAAGCCAGCCCCGACACCCGCCAACACCCGCTGACGCGCC
CTGACGGGCTTGTCTGCTCCCGGCATCCGCTTACAGACAAGCTGTGACCGTCTCCGGGAGCTGCATGTGTCAGAG
GTTTTCACCGTCATCACCGAAACGCGCGAGACGAAAGGGCCTCGTGATACGCCTATTTTTATAGGTTAATGTCATGAT
AATAATGGTTTCTTAGACGTCAGACGTCGCCCGCCGACAATAGGACGGCAAAGAACCGCTCCATGCCGCTCCTCGG
CGTAACAAGGAAGGAGCGCGATCCGGCAACGAAGCTCACAGCGAACATTGGTAACGCACTGCGAGAGCAAAACGA
TCGTCTTAGCCGTGCCGAAGAGATCGAGCGGCGTCTCGCTGAAGGTCAGGCAGTGATAGAGTTGGATGCCTCGTC
AATAGAACCGTCTTTCGTGCAGGATCGTATGCGAGGGGACATTGACGGGCTCCTTACTTCGATCCGGGAACAAGGA
CAGCAAGTCCCAATCCTTGTGCGACCGCATCCGAGCCAGCCGGGCCGATATCAGGTTGCCTTCGGCCACCGCCGG
CTACGCGCCGTTTCAGAACTCGGACTTCCGGTCAGGGCGGTCGTTCGCGAACTGACGGACGAGCAAGTGGTCGTA
GCACAGGGTCAGGAAAACAATGTGCGCGAAGATCTTACCTTCATCGAAAAGGCGCGCTTCGCACATCGCCTGAACA
GGCAGTTTTCTCGAGAGATTGTCATCGCCGCGATGTCGATCGACAAGAGCAATTTGTCCAAGATGCTTCTGCTCGTT
GACGCCCTCCCCTCTGAACTGACCGATGCTATTGGTGCCGCTCCTGGTGTTGGACGGCCGAGTTGGCAACAACTT
GCCGAGCTGATTGAGAAAGTTTCTTCACCGGCCGACGTGGCTAAATATGCTATGTCGGAGGAAGTTCAAGCGCTGC
CATCGGCAGAACGATTCAAGGCGGTGATCGCTAGTCTGAAGCCCAGTCGGGTTGCGCGTGGACTTCCCGAGGTCA
TGGCCACCCCAGACGGCACCAGAATTGCACAGGTGACGCAGAGCAAGGCCAAACTGGAAATCACGATTGACAGGA
AGGCGACGCCCGATTTTGCGACCTTCGTGCTCGATCATGTGCCAGCGCTGTATCAAGCGTACCACGCTGAGAACCA
ACGGAAACGGGGAGAGTAAACCGCAAAAGAAAAGAGCCCCCTCAACGTCGCCGTCGCGGAAGCCCTTCTGTCTCT
CTAGCGCGAACAGAATCGCATTTCCTCGAATCCTCGTCAAGAGTTTTTAGCGCCGTTTTGGTGAGCTGATTTCCTTT
GCCTGCTGAAAGGTGAAAGATGATGCAGACAGGAAGTGTAACGACGCCATTCGGGCGGCGGCCAATGACGCTTGC
GCTTGTGCGGCGCCAGACGGCGCTGGCCGATATCAAACAAGGCAAGACAGCGGACAAGTGGAAGGTCTTTAGGGA
CGCGTCCGCGGCCATGGAACTACTTGGAATCCAGTCCAACAGTCTTGCCGTCCTTGATGCGCTATTGAGCTTTCAC
CCGGAAACGGAGTTGCGTCAGGAGGCACAGCTGATCGTCTTCCCGTCGAATGCTCAGCTTGCCCTTCGGGCGCAT
GGGATGGCTGGCGCGACTTTGCGTAGGCACATCGCCATGCTCGTGGAGTCAGGCTTGATCGTCCGGAAGGATAGC
GCCAACGGAAAGCGTTACGCTCGTAAGGATGGCGCTGGTCAGATCGAGCGCGCGTTTGGCTTCGATTTGTCTCCG
CTTCTCGCGCGGTCCGAAGAGCTAGCGATGATGGCACAGCAGGTGATGGCCGATCGAGCAGCATTCAGGATGGCC
AAAGAAAGTCTGACGATTTGCCGACGGGACGTTCGGAAGCTAATTACGGCAGCTATGGAAGAGGGAGCGGAGGGC
GACTGGCAAGCTGTCGAGGAAGTCTATGTGGAACTTGTGGGTAGAATCCCACGCGCCCCGACGCTTGCTGATGTAG

AGTCAATTCTCGAAGAGATGTGGATGCTCCAGGAAGAGATAATCAACCGGTTGGAAATTAGAGACAATTCAGAAAATA

ATAGCACCAATGCTGCCCAGAGCGAGCAGCACATACAGAACTCAAAACCCGAATCCGTTAATGAACTTGAACCTCGC

TCTGAAAAGGAGCAGGGCGCTAAGCCGAGTGAAATAGACCGGGCAAGGAGCGAGCCGATAAAAGCGTTCCCCCTC

GGGATGATCCTGAAAGCGTGCCCGACCATTGGCAATTATGGGCCGAGCGGTGCGGTTGCTAGCTGGCGTGACCTC

ATGTCGGCTGCGGTGGTGGTTCGGTCTATGCTGGGGGTCAGCCCGTCGGCTTACCAAGACGCGTGTGAGGCAATG

GGACCGGAGAATGCGGCAGCAGCGATGGCGTGCATTTTGGAGCGAGCGAACTTCATCAATTCGCCTGGGGGCTAT

CTCCGAGATCTGACACGGCGGAGCGAACTCGGGAAGTTTTCACTTGGCCCGATGATAATGGCGCTCTTGAAGGCTA

GCGGGCAGGGGACGTTGCGGTTTGGCTAGAATTAGCGAGTATGGAGCAGGATGGTCTGTGGTCAGCTGACCACAG

ACCTAATAGGTTGAAAACATGAGCGTTTTTTGGATGATCGACAGACCATCCGATTCCCGGAGTACCAAGCGTGCTCT

GATGGGAGCGATAACATTACTCAACAAGCACGAAGGCCCCATGCCGATCGTTGATCGTGAAGGAGAGCCTGCTCTA

CATGCGGCGGTATTTTGCCGGCCGAGGCATGTAGTCGCGGAGCACTGCCTATTTACTGCCCTAGGCACAAACGTTG

ACTCTTGGATCGAGCTGGCAGACAAAGCAATAACCCACACAGAGGACGATTAATGGCTGACGAAGAGATCCAGAAT

CCGCCGGACGGTACTGCTGCTGCCGAAGTTGAGCCGGCTGCTCCTAGAGGTAGAAGAGCAAAGAAAGCACCAGC

CGAAACAGCCCGCACGGGATCGTTCAAATCCGTGAAGCCGAAAACCCGCGGCCTCAGCAACCGAGAAAAACTGGA

GAAGATCGGTCAAATCGAAGCTCAGGTCGCTGGCGGCGCAACCTTGAAGGACGCCGTTAAGATCGTGGGTATTTCC

GTTCAGACCTATTATCAATGGAAGAGAGCTGCGGTTCAACCTGTCTCACAGAATCCGGCCGTGTCTGTTTCAGTTGA

CGATGAACTCGGCGAGTTCATCCAACTCGAGGAGGAAAATCGGCGGCTCAGAAAGCTAGCTTGTGGCAGGATATAT

TGTGGTGTAAACAAATTGACGCTTAGACAACTTAATAACACATTGCGGACGTTTTTAATGTACTGGGGTGGTTTTTCTT

TTCACCAGTGAGACGGGCAACAGCTGATTGCCCTTCACCGCCTGGCCCTGAGAGAGTTGCAGCAAGCGGTCCACG

CTGGTTTGCCCCAGCAGGCGAAAATCCTGTTTGATGGTGGTTCCGAAATCGGCAAAATCCCTTATAAATCAAAAGAAT

AGCCCGAGATAGGGTTGAGTGTTGTTCCAGTTTGGAACAAGAGTCCACTATTAAAGAACGTGGACTCCAACGTCAAA

GGGCGAAAAACCGTCTATCAGGGCGATGGCCCAAATTCCCGATCTAGTAACATAGATGACACCGCGCGCGATAATTT

ATCCTAGTTTGCGCGCTATATTTTGTTTTCTATCGCGTATTAAATGTATAATTGCGGGACTCTAATCATAAAAACCCATCT

CATAAATAACGTCATGCATTACATGTTAATTATTACATGCTTAACGTAATTCAACAGAAATTATATGATAATCATCGCAAGA

CCGGCAACAGGATTCAATCTTAAGAAACTTTATTGCCAAATGTTTGAACGATCGGGGCCCGATCATATTGTCGCTCAG

GATCGTGGCGTTGTGCTTGTCGGCCGTTGCTGTCGTAATGATATCGGCACCTTCGACCGCCTGTTCCGCAGAGATC

CCGTGGGCGAAGAACTCCAGCATGAGATCCCCGCGCTGGAGGATCATCCAGCCGGCGTCCCGGAAAACGATTCCG

AAGCCCAACCTTTCATAGAAGGCGGCGGTGGAATCGAAATCTCGTGATGGCAGGTTGGGCGTCGCTTGGTCGGTC

ATTTCGAACCCCAGAGTCCCGCTCAGAAGAACTCGTCAAGAAGGCGATAGAAGGCGATGCGCTGCGAATCGGGAG

CGGCGATACCGTAAAGCACGAGGAAGCGGTCAGCCCATTCGCCGCCAAGCTCTTCAGCAATATCACGGGTAGCCAA

CGCTATGTCCTGATAGCGGTCCGCCACACCCAGCCGGCCACAGTCGATGAATCCAGAAAAGCGGCCATTTTCCACC

ATGATATTCGGCAAGCAGGCATCGCCATGGGTCACGACGAGATCATCGCCGTCGGGCATACGCGCCTTGAGCCTGG

CGAACAGTTCGGCTGGCGCGAGCCCCTGATGCTCTTCGTCCAGATCATCCTGATCGACAAGACCGGCTTCCATCCG

AGTACGTGCTCGCTCGATGCGATGTTTCGCTTGGTGGTCGAATGGGCAGGTAGCCGGATCAAGCGTATGCAGCCGC

CGCATTGCATCAGCCATGATGGATACTTTCTCGGCAGGAGCAAGGTGAGATGACAGGAGATCCTGCCCCGGCACTT

CGCCCAATAGCAGCCAGTCCCTTCCCGCTTCAGTGACAACGTCGAGCACAGCTGCGCAAGGAACGCCCGTCGTGG

CCAGCCACGATAGCCGCGCTGCCTCGTCCTGTAGTTCATTCAGGGCACCGGACAGGTCGGTCTTGACAAAAAGAA
CCGGGCGCCCCTGCGCTGACAGCCGGAACACGGCGGCATCAGAGCAGCCGATTGTCTGTTGTGCCCAGTCATAG
CCGAATAGCCTCTCCACCCAAGCGGCCGGAGAACCTGCGTGCAATCCATCTTGTTCAATCATGCGAAACGATCCAG
ATCCGGTGCAGATTATTTGGATTGAGAGTGAATATGAGACTCTAATTGGATACCGAGGGACTAGAAGACGTCGCCCG
CCGACAATAGGACGGCAAAGAACCGCTCCATGCCGCTCCTCGGCGTAACAAGGAAGGAGCGCGATCCGGCAACGA
AGCTCACAGCGAACATTGGTAACGCACTGCGAGAGCAAAACGATCGTCTTAGCCGTGCCGAAGAGATCGAGCGGC
GTCTCGCTGAAGGTCAGGCAGTGATAGAGTTGGATGCCTCGTCAATAGAACCGTCTTTCGTGCAGGATCGTATGCG
AGGGGACATTGACGGGCTCCTTACTTCGATCCGGGAACAAGGACAGCAAGTCCCAATCCTTGTGCGACCGCATCC
GAGCCAGCCGGGCCGATATCAGGTTGCCTTCGGCCACCGCCGGCTACGCGCCGTTTCAGAACTCGGACTTCCGGT
CAGGGCGGTCGTTCGCGAACTGACGGACGAGCAAGTGGTCGTAGCACAGGGTCAGGAAAACAATGTGCGCGAAG
ATCTTACCTTCATCGAAAAGGCGCGCTTCGCACATCGCCTGAACAGGCAGTTTTCTCGAGAGATTGTCATCGCCGCG
ATGTCGATCGACAAGAGCAATTTGTCCAAGATGCTTCTGCTCGTTGACGCCCTCCCCTCTGAACTGACCGATGCTAT
TGGTGCCGCTCCTGGTGTTGGACGGCCGAGTTGGCAACAACTTGCCGAGCTGATTGAGAAAGTTTCTTCACCGGC
CGACGTGGCTAAATATGCTATGTCGGAGGAAGTTCAAGCGCTGCCATCGGCAGAACGATTCAAGGCGGTGATCGCT
AGTCTGAAGCCCAGTCGGGTTGCGCGTGGACTTCCCGAGGTCATGGCCACCCCAGACGGCACCAGAATTGCACAG
GTGACGCAGAGCAAGGCCAAACTGGAAATCACGATTGACAGGAAGGCGACGCCCGATTTTGCGACCTTCGTGCTC
GATCATGTGCCAGCGCTGTATCAAGCGTACCACGCTGAGAACCAACGGAAACGGGGAGAGTAAACCGCAAAAGAAA
AGAGCCCCCTCAACGTCGCCGTCGCGGAAGCCCTTCTGTCTCTCTAGCGCGAACAGAATCGCATTTCCTCGAATCC
TCGTCAAGAGTTTTTAGCGCCGTTTTGGTGAGCTGATTTCCTTTGCCTGCTGAAAGGTGAAAGATGATGCAGACAGG
AAGTGTAACGACGCCATTCGGGCGGCGGCCAATGACGCTTGCGCTTGTGCGGCGCCAGACGGCGCTGGCCGATAT
CAAACAAGGCAAGACAGCGGACAAGTGGAAGGTCTTTAGGGACGCGTCCGCGGCCATGGAACTACTTGGAATCCA
GTCCAACAGTCTTGCCGTCCTTGATGCGCTATTGAGCTTTCACCCGGAAACGGAGTTGCGTCAGGAGGCACAGCTG
ATCGTCTTCCCGTCGAATGCTCAGCTTGCCCTTCGGGCGCATGGGATGGCTGGCGCGACTTTGCGTAGGCACATCG
CCATGCTCGTGGAGTCAGGCTTGATCGTCCGGAAGGATAGCGCCAACGGAAAGCGTTACGCTCGTAAGGATGGCG
CTGGTCAGATCGAGCGCGCGTTTGGCTTCGATTTGTCTCCGCTTCTCGCGCGGTCCGAAGAGCTAGCGATGATGG
CACAGCAGGTGATGGCCGATCGAGCAGCATTCAGGATGGCCAAAGAAAGTCTGACGATTTGCCGACGGGACGTTC
GGAAGCTAATTACGGCAGCTATGGAAGAGGGAGCGGAGGGCGACTGGCAAGCTGTCGAGGAAGTCTATGTGGAAC
TTGTGGGTAGAATCCCACGCGCCCCGACGCTTGCTGATGTAGAGTCAATTCTCGAAGAGATGTGGATGCTCCAGGA
AGAGATAATCAACCGGTTGGAAATTAGAGACAATTCAGAAAATAATAGCACCAATGCTGCCCAGAGCGAGCAGCACA
TACAGAACTCAAAACCCGAATCCGTTAATGAACTTGAACCTCGCTCTGAAAAGGAGCAGGGCGCTAAGCCGAGTGA
AATAGACCGGGCAAGGAGCGAGCCGATAAAAGCGTTCCCCCTCGGGATGATCCTGAAAGCGTGCCCGACCATTGG
CAATTATGGGCCGAGCGGTGCGGTTGCTAGCTGGCGTGACCTCATGTCGGCTGCGGTGGTGGTTCGGTCTATGCT
GGGGGTCAGCCCGTCGGCTTACCAAGACGCGTGTGAGGCAATGGGACCGGAGAATGCGGCAGCAGCGATGGCGT
GCATTTTGGAGCGAGCGAACTTCATCAATTCGCCTGGGGGCTATCTCCGAGATCTGACACGGCGGAGCGAACTCGG
GAAGTTTTCACTTGGCCCGATGATAATGGCGCTCTTGAAGGCTAGCGGGCAGGGGACGTTGCGGTTTGGCTAGAAT
TAGCGAGTATGGAGCAGGATGGTCTGTGGTCAGCTGACCACAGACCTAATAGGTTGAAAACATGAGCGTTTTTTGGA

TGATCGACAGACCATCCGATTCCCGGAGTACCAAGCGTGCTCTGATGGGAGCGATAACATTACTCAACAAGCACGAA
GGCCCCATGCCGATCGTTGATCGTGAAGGAGAGCCTGCTCTACATGCGGCGGTATTTTGCCGGCCGAGGCATGTAG
TCGCGGAGCACTGCCTATTTACTGCCCTAGGCACAAACGTTGACTCTTGGATCGAGCTGGCAGACAAAGCAATAAC
CCACACAGAGGACGATTAATGGCTGACGAAGAGATCCAGAATCCGCCGGACGGTACTGCTGCTGCCGAAGTTGAG
CCGGCTGCTCCTAGAGGTAGAAGAGCAAAGAAAGCACCAGCCGAAACAGCCCGCACGGGATCGTTCAAATCCGTG
AAGCCGAAAACCCGCGGCCTCAGCAACCGAGAAAAACTGGAGAAGATCGGTCAAATCGAAGCTCAGGTCGCTGGC
GGCGCAACCTTGAAGGACGCCGTTAAGATCGTGGGTATTTCCGTTCAGACCTATTATCAATGGAAGAGAGCTGCGGT
TCAACCTGTCTCACAGAATCCGGCCGTGTCTGTTTCAGTTGACGATGAACTCGGCGAGTTCATCCAACTCGAGGAG
GAAAATCGGCGGCTCAGAAAGCTAGCTTGTGGCAGGATATATTGTGGTGTAAACAAATTGACGCTTAGACAACTTAAT
AACACATTGCGGACGTTTTTAATGTACTGGGGTGGTTTTTCTTTTCACCAGTGAGACGGGCAACAGCTGATTGCCCT
TCACCGCCTGGCCCTGAGAGAGTTGCAGCAAGCGGTCCACGCTGGTTTGCCCCAGCAGGCGAAAATCCTGTTTGA
TGGTGGTTCCGAAATCGGCAAAATCCCTTATAAATCAAAAGAATAGCCCGAGATAGGGTTGAGTGTTGTTCCAGTTTG
GAACAAGAGTCCACTATTAAAGAACGTGGACTCCAACGTCAAAGGGCGAAAAACCGTCTATCAGGGCGATGGCCCA
AATTCCCGATCTAGTAACATAGATGACACCGCGCGCGATAATTTATCCTAGTTTGCGCGCTATATTTTGTTTTCTATCGC
GTATTAAATGTATAATTGCGGGACTCTAATCATAAAAACCCATCTCATAAATAACGTCATGCATTACATGTTAATTATTACA
TGCTTAACGTAATTCAACAGAAATTATATGATAATCATCGCAAGACCGGCAACAGGATTCAATCTTAAGAAACTTTATTG
CCAAATGTTTGAACGATCGGGGCCCGATCATATTGTCGCTCAGGATCGTGGCGTTGTGCTTGTCGGCCGTTGCTGT
CGTAATGATATCGGCACCTTCGACCGCCTGTTCCGCAGAGATCCCGTGGGCGAAGAACTCCAGCATGAGATCCCCG
CGCTGGAGGATCATCCAGCCGGCGTCCCGGAAAACGATTCCGAAGCCCAACCTTTCATAGAAGGCGGCGGTGGAA
TCGAAATCTCGTGATGGCAGGTTGGGCGTCGCTTGGTCGGTCATTTCGAACCCCAGAGTCCCGCTCAGAAGAACTC
GTCAAGAAGGCGATAGAAGGCGATGCGCTGCGAATCGGGAGCGGCGATACCGTAAAGCACGAGGAAGCGGTCAG
CCCATTCGCCGCCAAGCTCTTCAGCAATATCACGGGTAGCCAACGCTATGTCCTGATAGCGGTCCGCCACACCCAG
CCGGCCACAGTCGATGAATCCAGAAAAGCGGCCATTTTCCACCATGATATTCGGCAAGCAGGCATCGCCATGGGTC
ACGACGAGATCATCGCCGTCGGGCATACGCGCCTTGAGCCTGGCGAACAGTTCGGCTGGCGCGAGCCCCTGATG
CTCTTCGTCCAGATCATCCTGATCGACAAGACCGGCTTCCATCCGAGTACGTGCTCGCTCGATGCGATGTTTCGCTT
GGTGGTCGAATGGGCAGGTAGCCGGATCAAGCGTATGCAGCCGCCGCATTGCATCAGCCATGATGGATACTTTCTC
GGCAGGAGCAAGGTGAGATGACAGGAGATCCTGCCCCGGCACTTCGCCCAATAGCAGCCAGTCCCTTCCCGCTTC
AGTGACAACGTCGAGCACAGCTGCGCAAGGAACGCCCGTCGTGGCCAGCCACGATAGCCGCGCTGCCTCGTCCT
GTAGTTCATTCAGGGCACCGGACAGGTCGGTCTTGACAAAAAGAACCGGGCGCCCCTGCGCTGACAGCCGGAACA
CGGCGGCATCAGAGCAGCCGATTGTCTGTTGTGCCCAGTCATAGCCGAATAGCCTCTCCACCCAAGCGGCCGGAG
AACCTGCGTGCAATCCATCTTGTTCAATCATGCGAAACGATCCAGATCCGGTGCAGATTATTTGGATTGAGAGTGAAT
ATGAGACTCTAATTGGATACCGAGGGCGGGCGATTAAAGACGTCGCCCGCCGACAATAGGACGGCAAAGAACCGCT
CCATGCCGCTCCTCGGCGTAACAAGGAAGGAGCGCGATCCGGCAACGAAGCTCACAGCGAACATTGGTAACGCAC
TGCGAGAGCAAAACGATCGTCTTAGCCGTGCCGAAGAGATCGAGCGGCGTCTCGCTGAAGGTCAGGCAGTGATAG
AGTTGGATGCCTCGTCAATAGAACCGTCTTTCGTGCAGGATCGTATGCGAGGGGACATTGACGGGCTCCTTACTTC
GATCCGGGAACAAGGACAGCAAGTCCCAATCCTTGTGCGACCGCATCCGAGCCAGCCGGGCCGATATCAGGTTGC

CTTCGGCCACCGCCGGCTACGCGCCGTTTCAGAACTCGGACTTCCGGTCAGGGCGGTCGTTCGCGAACTGACGG
ACGAGCAAGTGGTCGTAGCACAGGGTCAGGAAAACAATGTGCGCGAAGATCTTACCTTCATCGAAAAGGCGCGCTT
CGCACATCGCCTGAACAGGCAGTTTTCTCGAGAGATTGTCATCGCCGCGATGTCGATCGACAAGAGCAATTTGTCC
AAGATGCTTCTGCTCGTTGACGCCCTCCCCTCTGAACTGACCGATGCTATTGGTGCCGCTCCTGGTGTTGGACGGC
CGAGTTGGCAACAACTTGCCGAGCTGATTGAGAAAGTTTCTTCACCGGCCGACGTGGCTAAATATGCTATGTCGGA
GGAAGTTCAAGCGCTGCCATCGGCAGAACGATTCAAGGCGGTGATCGCTAGTCTGAAGCCCAGTCGGGTTGCGCG
TGGACTTCCCGAGGTCATGGCCACCCCAGACGGCACCAGAATTGCACAGGTGACGCAGAGCAAGGCCAAACTGGA
AATCACGATTGACAGGAAGGCGACGCCCGATTTTGCGACCTTCGTGCTCGATCATGTGCCAGCGCTGTATCAAGCG
TACCACGCTGAGAACCAACGGAAACGGGGAGAGTAAACCGCAAAAGAAAAGAGCCCCCTCAACGTCGCCGTCGCG
GAAGCCCTTCTGTCTCTCTAGCGCGAACAGAATCGCATTTCCTCGAATCCTCGTCAAGAGTTTTTAGCGCCGTTTTG
GTGAGCTGATTTCCTTTGCCTGCTGAAAGGTGAAAGATGATGCAGACAGGAAGTGTAACGACGCCATTCGGGCGGC
GGCCAATGACGCTTGCGCTTGTGCGGCGCCAGACGGCGCTGGCCGATATCAAACAAGGCAAGACAGCGGACAAGT
GGAAGGTCTTTAGGGACGCGTCCGCGGCCATGGAACTACTTGGAATCCAGTCCAACAGTCTTGCCGTCCTTGATGC
GCTATTGAGCTTTCACCCGGAAACGGAGTTGCGTCAGGAGGCACAGCTGATCGTCTTCCCGTCGAATGCTCAGCTT
GCCCTTCGGGCGCATGGGATGGCTGGCGCGACTTTGCGTAGGCACATCGCCATGCTCGTGGAGTCAGGCTTGATC
GTCCGGAAGGATAGCGCCAACGGAAAGCGTTACGCTCGTAAGGATGGCGCTGGTCAGATCGAGCGCGCGTTTGGC
TTCGATTTGTCTCCGCTTCTCGCGCGGTCCGAAGAGCTAGCGATGATGGCACAGCAGGTGATGGCCGATCGAGCA
GCATTCAGGATGGCCAAAGAAAGTCTGACGATTTGCCGACGGGACGTTCGGAAGCTAATTACGGCAGCTATGGAAG
AGGGAGCGGAGGGCGACTGGCAAGCTGTCGAGGAAGTCTATGTGGAACTTGTGGGTAGAATCCCACGCGCCCCG
ACGCTTGCTGATGTAGAGTCAATTCTCGAAGAGATGTGGATGCTCCAGGAAGAGATAATCAACCGGTTGGAAATTAG
AGACAATTCAGAAAATAATAGCACCAATGCTGCCCAGAGCGAGCAGCACATACAGAACTCAAAACCCGAATCCGTTA
ATGAACTTGAACCTCGCTCTGAAAAGGAGCAGGGCGCTAAGCCGAGTGAAATAGACCGGGCAAGGAGCGAGCCGA
TAAAAGCGTTCCCCCTCGGGATGATCCTGAAAGCGTGCCCGACCATTGGCAATTATGGGCCGAGCGGTGCGGTTGC
TAGCTGGCGTGACCTCATGTCGGCTGCGGTGGTGGTTCGGTCTATGCTGGGGGTCAGCCCGTCGGCTTACCAAGA
CGCGTGTGAGGCAATGGGACCGGAGAATGCGGCAGCAGCGATGGCGTGCATTTTGGAGCGAGCGAACTTCATCAA
TTCGCCTGGGGGCTATCTCCGAGATCTGACACGGCGGAGCGAACTCGGGAAGTTTTCACTTGGCCCGATGATAATG
GCGCTCTTGAAGGCTAGCGGGCAGGGGACGTTGCGGTTTGGCTAGAATTAGCGAGTATGGAGCAGGATGGTCTGT
GGTCAGCTGACCACAGACCTAATAGGTTGAAAACATGAGCGTTTTTTGGATGATCGACAGACCATCCGATTCCCGGA
GTACCAAGCGTGCTCTGATGGGAGCGATAACATTACTCAACAAGCACGAAGGCCCCATGCCGATCGTTGATCGTGA
AGGAGAGCCTGCTCTACATGCGGCGGTATTTTGCCGGCCGAGGCATGTAGTCGCGGAGCACTGCCTATTTACTGCC
CTAGGCACAAACGTTGACTCTTGGATCGAGCTGGCAGACAAAGCAATAACCCACACAGAGGACGATTAATGGCTGA
CGAAGAGATCCAGAATCCGCCGGACGGTACTGCTGCTGCCGAAGTTGAGCCGGCTGCTCCTAGAGGTAGAAGAGC
AAAGAAAGCACCAGCCGAAACAGCCCGCACGGGATCGTTCAAATCCGTGAAGCCGAAAACCCGCGGCCTCAGCAA
CCGAGAAAAACTGGAGAAGATCGGTCAAATCGAAGCTCAGGTCGCTGGCGGCGCAACCTTGAAGGACGCCGTTAA
GATCGTGGGTATTTCCGTTCAGACCTATTATCAATGGAAGAGAGCTGCGGTTCAACCTGTCTCACAGAATCCGGCCG
TGTCTGTTTCAGTTGACGATGAACTCGGCGAGTTCATCCAACTCGAGGAGGAAAATCGGCGGCTCAGAAAGCTAGC

TTGTGGCAGGATATATTGTGGTGTAAACAAATTGACGCTTAGACAACTTAATAACACATTGCGGACGTTTTTAATGTAC
TGGGGTGGTTTTTCTTTTCACCAGTGAGACGGGCAACAGCTGATTGCCCTTCACCGCCTGGCCCTGAGAGAGTTGC
AGCAAGCGGTCCACGCTGGTTTGCCCCAGCAGGCGAAAATCCTGTTTGATGGTGGTTCCGAAATCGGCAAAATCCC
TTATAAATCAAAAGAATAGCCCGAGATAGGGTTGAGTGTTGTTCCAGTTTGGAACAAGAGTCCACTATTAAAGAACGT
GGACTCCAACGTCAAAGGGCGAAAAACCGTCTATCAGGGCGATGGCCCAAATTCCCGATCTAGTAACATAGATGACA
CCGCGCGCGATAATTTATCCTAGTTTGCGCGCTATATTTTGTTTTCTATCGCGTATTAAATGTATAATTGCGGGACTCTA
ATCATAAAAACCCATCTCATAAATAACGTCATGCATTACATGTTAATTATTACATGCTTAACGTAATTCAACAGAAATTATA
TGATAATCATCGCAAGACCGGCAACAGGATTCAATCTTAAGAAACTTTATTGCCAAATGTTTGAACGATCGGGGCCCG
ATCATATTGTCGCTCAGGATCGTGGCGTTGTGCTTGTCGGCCGTTGCTGTCGTAATGATATCGGCACCTTCGACCGC
CTGTTCCGCAGAGATCCCGTGGGCGAAGAACTCCAGCATGAGATCCCCGCGCTGGAGGATCATCCAGCCGGCGTC
CCGGAAAACGATTCCGAAGCCCAACCTTTCATAGAAGGCGGCGGTGGAATCGAAATCTCGTGATGGCAGGTTGGG
CGTCGCTTGGTCGGTCATTTCGAACCCCAGAGTCCCGCTCAGAAGAACTCGTCAAGAAGGCGATAGAAGGCGATG
CGCTGCGAATCGGGAGCGGCGATACCGTAAAGCACGAGGAAGCGGTCAGCCCATTCGCCGCCAAGCTCTTCAGCA
ATATCACGGGTAGCCAACGCTATGTCCTGATAGCGGTCCGCCACACCCAGCCGGCCACAGTCGATGAATCCAGAAA
AGCGGCCATTTTCCACCATGATATTCGGCAAGCAGGCATCGCCATGGGTCACGACGAGATCATCGCCGTCGGGCAT
ACGCGCCTTGAGCCTGGCGAACAGTTCGGCTGGCGCGAGCCCCTGATGCTCTTCGTCCAGATCATCCTGATCGAC
AAGACCGGCTTCCATCCGAGTACGTGCTCGCTCGATGCGATGTTTCGCTTGGTGGTCGAATGGGCAGGTAGCCGG
ATCAAGCGTATGCAGCCGCCGCATTGCATCAGCCATGATGGATACTTTCTCGGCAGGAGCAAGGTGAGATGACAGG
AGATCCTGCCCCGGCACTTCGCCCAATAGCAGCCAGTCCCTTCCCGCTTCAGTGACAACGTCGAGCACAGCTGCG
CAAGGAACGCCCGTCGTGGCCAGCCACGATAGCCGCGCTGCCTCGTCCTGTAGTTCATTCAGGGCACCGGACAGG
TCGGTCTTGACAAAAAGAACCGGGCGCCCCTGCGCTGACAGCCGGAACACGGCGGCATCAGAGCAGCCGATTGT
CTGTTGTGCCCAGTCATAGCCGAATAGCCTCTCCACCCAAGCGGCCGGAGAACCTGCGTGCAATCCATCTTGTTCA
ATCATGCGAAACGATCCAGATCCGGTGCAGATTATTTGGATTGAGAGTGAATATGAGACTCTAATTGGATACCGAGGG
CGGGCGATCGCAGACGTCGCCCGCCGACAATAGGACGGCAAAGAACCGCTCCATGCCGCTCCTCGGCGTAACAA
GGAAGGAGCGCGATCCGGCAACGAAGCTCACAGCGAACATTGGTAACGCACTGCGAGAGCAAAACGATCGTCTTA
GCCGTGCCGAAGAGATCGAGCGGCGTCTCGCTGAAGGTCAGGCAGTGATAGAGTTGGATGCCTCGTCAATAGAAC
CGTCTTTCGTGCAGGATCGTATGCGAGGGGACATTGACGGGCTCCTTACTTCGATCCGGGAACAAGGACAGCAAGT
CCCAATCCTTGTGCGACCGCATCCGAGCCAGCCGGGCCGATATCAGGTTGCCTTCGGCCACCGCCGGCTACGCGC
CGTTTCAGAACTCGGACTTCCGGTCAGGGCGGTCGTTCGCGAACTGACGGACGAGCAAGTGGTCGTAGCACAGG
GTCAGGAAAACAATGTGCGCGAAGATCTTACCTTCATCGAAAAGGCGCGCTTCGCACATCGCCTGAACAGGCAGTT
TTCTCGAGAGATTGTCATCGCCGCGATGTCGATCGACAAGAGCAATTTGTCCAAGATGCTTCTGCTCGTTGACGCCC
TCCCCTCTGAACTGACCGATGCTATTGGTGCCGCTCCTGGTGTTGGACGGCCGAGTTGGCAACAACTTGCCGAGCT
GATTGAGAAAGTTTCTTCACCGGCCGACGTGGCTAAATATGCTATGTCGGAGGAAGTTCAAGCGCTGCCATCGGCA
GAACGATTCAAGGCGGTGATCGCTAGTCTGAAGCCCAGTCGGGTTGCGCGTGGACTTCCCGAGGTCATGGCCACC
CCAGACGGCACCAGAATTGCACAGGTGACGCAGAGCAAGGCCAAACTGGAAATCACGATTGACAGGAAGGCGACG
CCCGATTTTGCGACCTTCGTGCTCGATCATGTGCCAGCGCTGTATCAAGCGTACCACGCTGAGAACCAACGGAAAC

GGGGAGAGTAAACCGCAAAAGAAAAGAGCCCCCTCAACGTCGCCGTCGCGGAAGCCCTTCTGTCTCTCTAGCGCG
AACAGAATCGCATTTCCTCGAATCCTCGTCAAGAGTTTTTAGCGCCGTTTTGGTGAGCTGATTTCCTTTGCCTGCTGA
AAGGTGAAAGATGATGCAGACAGGAAGTGTAACGACGCCATTCGGGCGGCGGCCAATGACGCTTGCGCTTGTGCG
GCGCCAGACGGCGCTGGCCGATATCAAACAAGGCAAGACAGCGGACAAGTGGAAGGTCTTTAGGGACGCGTCCG
CGGCCATGGAACTACTTGGAATCCAGTCCAACAGTCTTGCCGTCCTTGATGCGCTATTGAGCTTTCACCCGGAAACG
GAGTTGCGTCAGGAGGCACAGCTGATCGTCTTCCCGTCGAATGCTCAGCTTGCCCTTCGGGCGCATGGGATGGCT
GGCGCGACTTTGCGTAGGCACATCGCCATGCTCGTGGAGTCAGGCTTGATCGTCCGGAAGGATAGCGCCAACGGA
AAGCGTTACGCTCGTAAGGATGGCGCTGGTCAGATCGAGCGCGCGTTTGGCTTCGATTTGTCTCCGCTTCTCGCGC
GGTCCGAAGAGCTAGCGATGATGGCACAGCAGGTGATGGCCGATCGAGCAGCATTCAGGATGGCCAAAGAAAGTC
TGACGATTTGCCGACGGGACGTTCGGAAGCTAATTACGGCAGCTATGGAAGAGGGAGCGGAGGGCGACTGGCAAG
CTGTCGAGGAAGTCTATGTGGAACTTGTGGGTAGAATCCCACGCGCCCCGACGCTTGCTGATGTAGAGTCAATTCT
CGAAGAGATGTGGATGCTCCAGGAAGAGATAATCAACCGGTTGGAAATTAGAGACAATTCAGAAAATAATAGCACCA
ATGCTGCCCAGAGCGAGCAGCACATACAGAACTCAAAACCCGAATCCGTTAATGAACTTGAACCTCGCTCTGAAAAG
GAGCAGGGCGCTAAGCCGAGTGAAATAGACCGGGCAAGGAGCGAGCCGATAAAAGCGTTCCCCCTCGGGATGATC
CTGAAAGCGTGCCCGACCATTGGCAATTATGGGCCGAGCGGTGCGGTTGCTAGCTGGCGTGACCTCATGTCGGCT
GCGGTGGTGGTTCGGTCTATGCTGGGGGTCAGCCCGTCGGCTTACCAAGACGCGTGTGAGGCAATGGGACCGGA
GAATGCGGCAGCAGCGATGGCGTGCATTTTGGAGCGAGCGAACTTCATCAATTCGCCTGGGGGCTATCTCCGAGAT
CTGACACGGCGGAGCGAACTCGGGAAGTTTTCACTTGGCCCGATGATAATGGCGCTCTTGAAGGCTAGCGGGCAG
GGGACGTTGCGGTTTGGCTAGAATTAGCGAGTATGGAGCAGGATGGTCTGTGGTCAGCTGACCACAGACCTAATAG
GTTGAAAACATGAGCGTTTTTTGGATGATCGACAGACCATCCGATTCCCGGAGTACCAAGCGTGCTCTGATGGGAG
CGATAACATTACTCAACAAGCACGAAGGCCCCATGCCGATCGTTGATCGTGAAGGAGAGCCTGCTCTACATGCGGC
GGTATTTTGCCGGCCGAGGCATGTAGTCGCGGAGCACTGCCTATTTACTGCCCTAGGCACAAACGTTGACTCTTGG
ATCGAGCTGGCAGACAAAGCAATAACCCACACAGAGGACGATTAATGGCTGACGAAGAGATCCAGAATCCGCCGGA
CGGTACTGCTGCTGCCGAAGTTGAGCCGGCTGCTCCTAGAGGTAGAAGAGCAAAGAAAGCACCAGCCGAAACAGC
CCGCACGGGATCGTTCAAATCCGTGAAGCCGAAAACCCGCGGCCTCAGCAACCGAGAAAAACTGGAGAAGATCGG
TCAAATCGAAGCTCAGGTCGCTGGCGGCGCAACCTTGAAGGACGCCGTTAAGATCGTGGGTATTTCCGTTCAGACC
TATTATCAATGGAAGAGAGCTGCGGTTCAACCTGTCTCACAGAATCCGGCCGTGTCTGTTTCAGTTGACGATGAACT
CGGCGAGTTCATCCAACTCGAGGAGGAAAATCGGCGGCTCAGAAAGCTAGCTTGTGGCAGGATATATTGTGGTGTA
AACAAATTGACGCTTAGACAACTTAATAACACATTGCGGACGTTTTTAATGTACTGGGGTGGTTTTTCTTTTCACCAGT
GAGACGGGCAACAGCTGATTGCCCTTCACCGCCTGGCCCTGAGAGAGTTGCAGCAAGCGGTCCACGCTGGTTTGC
CCCAGCAGGCGAAAATCCTGTTTGATGGTGGTTCCGAAATCGGCAAAATCCCTTATAAATCAAAAGAATAGCCCGAG
ATAGGGTTGAGTGTTGTTCCAGTTTGGAACAAGAGTCCACTATTAAAGAACGTGGACTCCAACGTCAAAGGGCGAAA
AACCGTCTATCAGGGCGATGGCCCAAATTCCCGATCTAGTAACATAGATGACACCGCGCGCGATAATTTATCCTAGTT
TGCGCGCTATATTTTGTTTTCTATCGCGTATTAAATGTATAATTGCGGGACTCTAATCATAAAAACCCATCTCATAAATAA
CGTCATGCATTACATGTTAATTATTACATGCTTAACGTAATTCAACAGAAATTATATGATAATCATCGCAAGACCGGCAA
CAGGATTCAATCTTAAGAAACTTTATTGCCAAATGTTTGAACGATCGGGGCCCGATCATATTGTCGCTCAGGATCGTG

GCGTTGTGCTTGTCGGCCGTTGCTGTCGTAATGATATCGGCACCTTCGACCGCCTGTTCCGCAGAGATCCCGTGGG
CGAAGAACTCCAGCATGAGATCCCCGCGCTGGAGGATCATCCAGCCGGCGTCCCGGAAAACGATTCCGAAGCCCA
ACCTTTCATAGAAGGCGGCGGTGGAATCGAAATCTCGTGATGGCAGGTTGGGCGTCGCTTGGTCGGTCATTTCGAA
CCCCAGAGTCCCGCTCAGAAGAACTCGTCAAGAAGGCGATAGAAGGCGATGCGCTGCGAATCGGGAGCGGCGATA
CCGTAAAGCACGAGGAAGCGGTCAGCCCATTCGCCGCCAAGCTCTTCAGCAATATCACGGGTAGCCAACGCTATGT
CCTGATAGCGGTCCGCCACACCCAGCCGGCCACAGTCGATGAATCCAGAAAAGCGGCCATTTTCCACCATGATATT
CGGCAAGCAGGCATCGCCATGGGTCACGACGAGATCATCGCCGTCGGGCATACGCGCCTTGAGCCTGGCGAACA
GTTCGGCTGGCGCGAGCCCCTGATGCTCTTCGTCCAGATCATCCTGATCGACAAGACCGGCTTCCATCCGAGTACG
TGCTCGCTCGATGCGATGTTTCGCTTGGTGGTCGAATGGGCAGGTAGCCGGATCAAGCGTATGCAGCCGCCGCATT
GCATCAGCCATGATGGATACTTTCTCGGCAGGAGCAAGGTGAGATGACAGGAGATCCTGCCCCGGCACTTCGCCCA
ATAGCAGCCAGTCCCTTCCCGCTTCAGTGACAACGTCGAGCACAGCTGCGCAAGGAACGCCCGTCGTGGCCAGCC
ACGATAGCCGCGCTGCCTCGTCCTGTAGTTCATTCAGGGCACCGGACAGGTCGGTCTTGACAAAAAGAACCGGGC
GCCCCTGCGCTGACAGCCGGAACACGGCGGCATCAGAGCAGCCGATTGTCTGTTGTGCCCAGTCATAGCCGAATA
GCCTCTCCACCCAAGCGGCCGGAGAACCTGCGTGCAATCCATCTTGTTCAATCATGCGAAACGATCCAGATCCGGT
GCAGATTATTTGGATTGAGAGTGAATATGAGACTCTAATTGGATACCGAGGGCGGTTAATTAAGCACTTGTCGACTCT
AGAGGATCCCCGGGTACCGAGCTCGAATTCGTAATCATGGTCATAGCTGTTTCCTGTGTGAAATTGTTATCCGCTCAC
AATTCCACACAACATACGAGCCGGAAGCATAAAGTGTAAAGCCTGGGGTGCCTAATGAGTGAGCTAACTCACATTAC
TTGGCAGAACATATCCATCGCGTCCGCCATCTCCAGCAGCCGCACGCGGCGCATCTCGGGCAGCGTTGGGTCCTG
GCCACGGGTGCGCATGATCGTGCTCCTGTCGTTGAGGACCCGGCTAGGCTGGCGGGGTTGCCTTACTGGTTAGCA
GAATGAATCACCGATACGCGAGCGAACGTGAAGCGACTGCTGCTGCAAAACGTCTGCGACCTGAGCAACAACATGA
ATGGTCTTCGGTTTCCGTGTTTCGTAAAGTCTGGAAACGCGGAAGTCAGCGCCCTGCACCATTATGTTCCGGATCTG
CATCGCAGGATGCTGCTGGCTACCCTGTGGAACACCTACATCTGTATTAACGAAGCGCTGGCATTGACCCTGAGTGA
TTTTTCTCTGGTCCCGCCGCATCCATACCGCCAGTTGTTTACCCTCACAACGTTCCAGTAACCGGGCATGTTCATCAT
CAGTAACCCGTATCGTGAGCATCCTCTCTCGTTTCATCGGTATCATTACCCCCATGAACAGAAATCCCCCTTACACGG
AGGCATCAGTGACCAAACAGGAAAAAACCGCCCTTAACATGGCCCGCTTTATCAGAAGCCAGACATTAACGCTTCTG
GAGAAACTCAACGAGCTGGACGCGGATGAACAGGCAGACATCTGTGAATCGCTTCACGACCACGCTGATGAGCTTT
ACCGCAGCTGCCTCGCGCGTTTCGGTGATGACGGTGAAAACCTCTGACACATGCAGCTCCCGGAGACGGTCACAG
CTTGTCTGTAAGCGGATGCCGGGAGCAGACAAGCCCGTCAGGGCGCGTCAGCGGGTGTTGGCGGGTGTCGGGG
CGCAGCCATGACCCAGTCACGTAGCGATAGCGGAGTGTATACTGGCTTAACTATGCGGCATCAGAGCAGATTGTACT
GAGAGTGCACCATATGCGGTGTGAAATACCGCACAGATGCGTAAGGAGAAAATACCGCATCAGGCGCTCTTCCGCT
TCCTCGCTCACTGACTCGCTGCGCTCGGTCGTTCGGCTGCGGCGAGCGGTATCAGCTCACTCAAAGGCGGTAATA
CGGTTATCCACAGAATCAGGGGATAACGCAGGAAAGAACATGTGAGCAAAAGGCCAGCAAAAGGCCAGGAACCGTA
AAAAGGCCGCGTTGCTGGCGTTTTTCCATAGGCTCCGCCCCCCTGACGAGCATCACAAAAATCGACGCTCAAGTCA
GAGGTGGCGAAACCCGACAGGACTATAAAGATACCAGGCGTTTCCCCCTGGAAGCTCCCTCGTGCGCTCTCCTGTT
CCGACCCTGCCGCTTACCGGATACCTGTCCGCCTTTCTCCCTTCGGGAAGCGTGGCGCTTTCTCATAGCTCACGCT
GTAGGTATCTCAGTTCGGTGTAGGTCGTTCGCTCCAAGCTGGGCTGTGTGCACGAACCCCCCGTTCAGCCCGACC

GCTGCGCCTTATCCGGTAACTATCGTCTTGAGTCCAACCCGGTAAGACACGACTTATCGCCACTGGCAGCAGCCAC
TGGTAACAGGATTAGCAGAGCGAGGTATGTAGGCGGTGCTACAGAGTTCTTGAAGTGGTGGCCTAACTACGGCTAC
ACTAGAAGGACAGTATTTGGTATCTGCGCTCTGCTGAAGCCAGTTACCTTCGGAAAAAGAGTTGGTAGCTCTTGATC
CGGCAAACAAACCACCGCTGGTAGCGGTGGTTTTTTTGTTTGCAAGCAGCAGATTACGCGCAGAAAAAAAGGATCT
CAAGAAGATCCTTTGATCTTTTCTACGGGGTCTGACGCTCAGTGGAACGAAAACTCACGTTAAGGGATTTTGGTCAT
GAGATTATCAAAAAGGATCTTCACCTAGATCCTTTTAAATTAAAAATGAAGTTTTAAATCAATCTAAAGTATATATGAGTA
AACTTGGTCTGACAGTTACCAATGCTTAATCAGTGAGGCACCTATCTCAGCGATCTGTCTATTTCGTTCATCCATAGTT
GCCTGACTCCCCGTCGTGTAGATAACTACGATACGGGAGGGCTTACCATCTGGCCCCAGTGCTGCAATGATACCGC
GAGACCCACGCTCACCGGCTCCAGATTTATCAGCAATAAACCAGCCAGCCGGAAGGGCCGAGCGCAGAAGTGGTC
CTGCAACTTTATCCGCCTCCATCCAGTCTATTAATTGTTGCCGGGAAGCTAGAGTAAGTAGTTCGCCAGTTAATAGTTT
GCGCAACGTTGTTGCCATTGCTACAGGCATCGTGGTGTCACGCTCGTCGTTTGGTATGGCTTCATTCAGCTCCGGTT
CCCAACGATCAAGGCGAGTTACATGATCCCCCATGTTGTGCAAAAAAGCGGTTAGCTCCTTCGGTCCTCCGATCGTT
GTCAGAAGTAAGTTGGCCGCAGTGTTATCACTCATGGTTATGGCAGCACTGCATAATTCTCTTACTGTCATGCCATCC
GTAAGATGCTTTTCTGTGACTGGTGAGTACTCAACCAAGTCATTCTGAGAATAGTGTATGCGGCGACCGAGTTGCTC
TTGCCCGGCGTCAATACGGGATAATACCGCGCCACATAGCAGAACTTTAAAAGTGCTCATCATTGGAAAACGTTCTTC
GGGGCGAAAACTCTCAAGGATCTTACCGCTGTTGAGATCCAGTTCGATGTAACCCACTCGTGCACCCAACTGATCTT
CAGCATCTTTTACTTTCACCAGCGTTTCTGGGTGAGCAAAAACAGGAAGGCAAAATGCCGCAAAAAAGGGAATAAGG
GCGACACGGAAATGTTGAATACTCATACTCTTCTTTTTCAATATTATTGAAGCATTTATCAGGGTTATTGTCTCATGAG
CGGATACATATTTGAATGTATTTAGAAAAATAAACAAATAGGGGTTCCGCGCACATTTCCCCGAAAAGTGCCACCTGA
CGTCTAAGAAACCATTATTATCATGACATTAACCTATAAAAATAGGCGTATCACGAGGCCCTTTCGTC
